# Can Point-of-Care Urine LAM Strip Testing for Tuberculosis Add Value to Clinical Decision Making in Hospitalised HIV-Infected Persons?

**DOI:** 10.1371/journal.pone.0054875

**Published:** 2013-02-04

**Authors:** Jonathan G. Peter, Grant Theron, Keertan Dheda

**Affiliations:** 1 Lung Infection and Immunity Unit, Division of Pulmonology & UCT Lung Institute, Department of Medicine, University of Cape Town, Cape Town, South Africa; 2 Institute of Infectious Diseases and Molecular Medicine, University of Cape Town, Cape Town, South Africa; 3 Department of Infection, University College London Medical School, London, United Kingdom; San Francisco General Hospital, University of California San Francisco, United States of America

## Abstract

**Background:**

The urine lipoarabinomannan (LAM) strip-test (Determine®-TB) can rapidly rule-in TB in HIV-infected persons with advanced immunosuppression. However, given high rates of empiric treatment amongst hospitalised patients in high-burden settings (∼50%) it is unclear whether LAM can add any value to clinical decision making, or identify a subset of patients with unfavourable outcomes that would otherwise have been missed by empiric treatment.

**Methods:**

281 HIV-infected hospitalised patients with suspected TB received urine LAM strip testing, and were categorised as definite (culture-positive), probable-, or non-TB. Both the proportion and morbidity of TB cases identified by LAM testing, early empiric treatment (initiated prior to test result availability) and a set of clinical predictors were compared across groups.

**Results:**

187/281 patients had either definite- (n = 116) or probable-TB (n = 71). As a rule-in test for definite and probable-TB, LAM identified a similar proportion of TB cases compared to early empiric treatment (85/187 vs. 93/187, p = 0.4), but a greater proportion than classified by a set of clinical predictors alone (19/187; p<0.001). Thirty-nine of the 187 (21%) LAM-positive patients who had either definite- or probable-TB were missed by early empiric treatment, and of these 25/39 (64%) would also have been missed by smear microscopy. Thus, 25/187 (8%) of definite- or probable-TB patients with otherwise delayed initiation of TB treatment could be detected by the LAM strip test. LAM-positive patients missed by early empiric treatment had a lower median CD4 count (p = 0.008), a higher median illness severity score (p = 0.001) and increased urea levels (p = 0.002) compared to LAM-negative patients given early empiric treatment.

**Conclusions:**

LAM strip testing outperformed TB diagnosis based on clinical criteria but in day-to-day practice identified a similar proportion of patients compared to early empiric treatment. However, compared to empiric treatment, LAM identified a different subset of patients with more advanced immunosuppression and greater disease severity.

## Introduction

The early high mortality (>25%) amongst hospitalised TB HIV co-infected patients in resource-poor settings requires urgent attention [1], [2]. The increased incidence of sputum pauci-bacillary, and disseminated forms of TB in these patients limits the use of both traditional and new TB diagnostic tools [3]–[6]. Empiric TB treatment, based only on clinical and radiological findings is common (∼50%) amongst hospitalised HIV-infected patients with advanced immunosuppression; given their high pre-test probability of disease and illness severity [7], [8]. Formalized World Health Organisation (WHO) clinical algorithms are available to guide empiric treatment and, despite modest diagnostic accuracy in ambulatory patients [9], [10], evidence suggests that their use may reduce mortality amongst hospitalised HIV-infected patients [8]. Although death due to undiagnosed TB is common in hospitalised HIV-infected patients in Africa [2], [8], empiric treatment guidelines and practices are inconsistent, vary between hospitals, and may needlessly expose patients to toxic drugs. Thus, there remains a need for simple bedside tools to help guide the early initiation of TB treatment, where a microbiological diagnosis may be unavailable or delayed.

Urinary lipoarabinomannan (LAM) has more recently been evaluated for the diagnosis of TB in HIV-infected patients [11], [12]. In hospitalised HIV-infected patients, a urinary LAM ELISA (Alere, USA) has an overall sensitivity of 59–67%, increasing to as high as 85% in patients with CD4<50 cells/ml, and an overall specificity of 80–94% [13]–[15]. In addition, LAM positivity has been associated with higher mycobacterial burden, more severe illness, and a higher mortality [15]–[17]. As an alternative to the ELISA-kit, LAM can now be detected by a simple, low-cost (<US$3.5) point-of-care lateral flow assay that is able to provide results in 25 min from just 60 µl of urine [11], [12]. Initial evaluation studies have confirmed equivalent performance of the urine LAM strip test compared to the LAM ELISA assay in different settings [13], [18].

However, is this test really useful in ‘real world’ clinical practice in high HIV prevalence settings? The real value in any diagnostic lies in its ability to provide information beyond that deducible from basic clinical and radiographic data, such that it adds incremental value to routine clinical practice. Useful tests add value to clinical decision-making by ruling-in patients not otherwise routinely identifiable, pinpointing otherwise unrecognizable patients with the highest risk of morbidity and mortality, or by reducing unnecessary treatment. In this context, our study investigated whether point-of-care urine LAM strip testing offered any value over basic clinical and radiological screening, and whether testing was redundant in the context of routine ‘real world’ day-to-day clinical practice where empiric treatment is commonly used. We therefore evaluated LAM strip test performance against physician-led empiric treatment decisions and a set of clinical predictors.

## Methods

### Study Population

**Figure 1:**
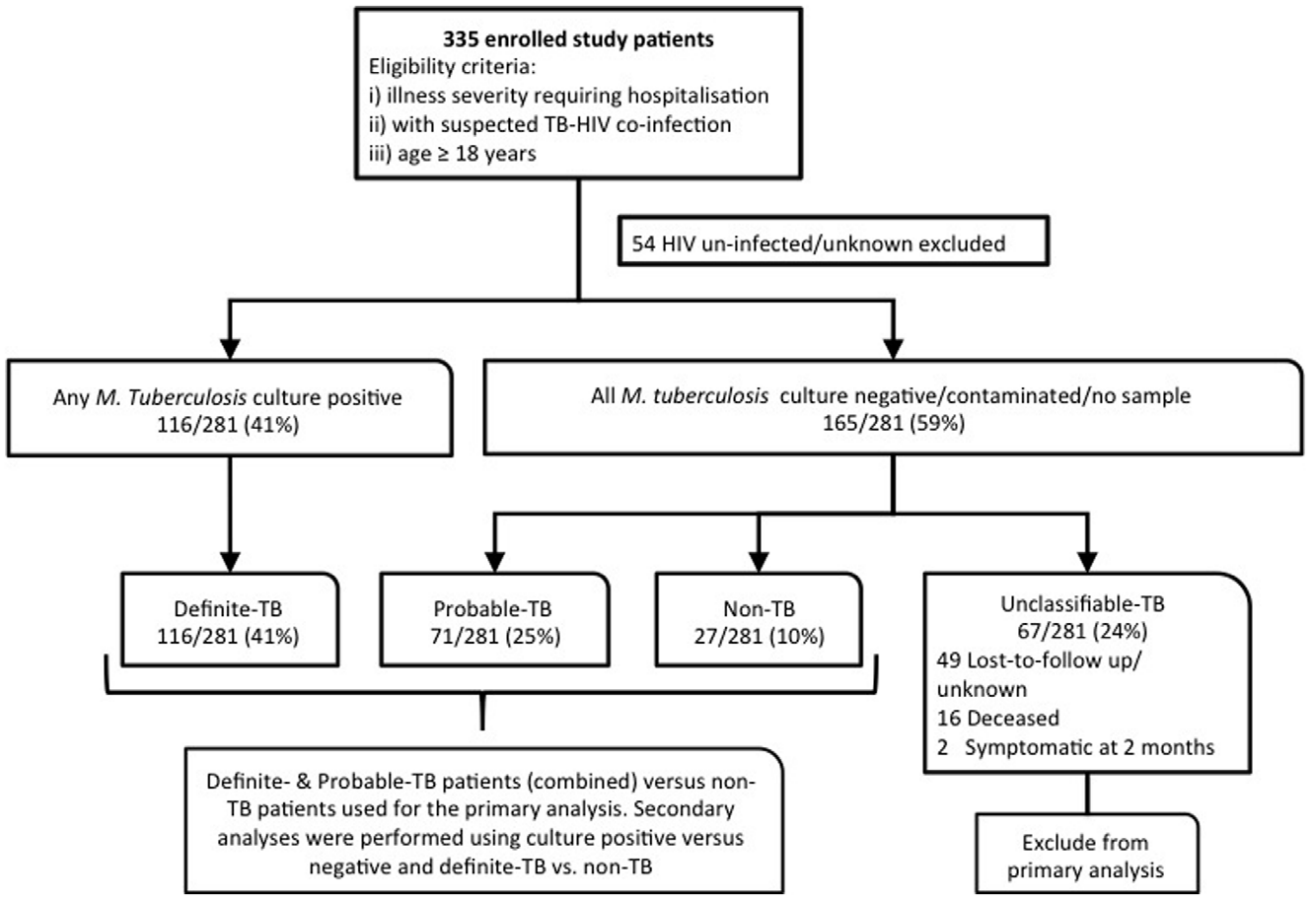
Study population, culture status and diagnostic groups used for analyses.

A study outline is shown in Figure 1. In total, 335 prospectively recruited adult in-patients patients from four hospitals (three district- and one tertiary-level) between July 2009 and December 2010 in Cape Town, South Africa were enrolled. Patients were referred for study inclusion by emergency-room or clinic doctors if suspected to have HIV-TB co-infection and needed in-patient care. All patients provided written informed consent and the University of Cape Town Faculty of Health Sciences Human Research Ethics Committee approved the study. Clinical information collected included: demographics, past history of TB, co-morbidity, symptoms, vital signs (including weight) and a modified early warning (MEWS) illness severity score [19]. Blood was taken for HIV, CD4 and renal function testing. A chest radiograph (CXR) was performed in all patients.

### TB Diagnostic Sampling and Testing

Consultant-led hospital-based clinicians not associated with the study team determined the timing and extent of TB diagnostic work-up and the commencement of empiric anti-TB treatment. Routine hospital practice includes the collection, where possible, of two sputum samples in patients able to expectorate and, if extra-pulmonary TB is suspected, the collection of 1–2 non-sputum samples from clinically involved sites (e.g. fine needle aspirate of lymph node, pleural fluid aspirate/biopsy, ascitic tap, lumbar puncture, pericardial aspiration etc.). Further details of biological samples collected for TB culture, stratified by patient TB diagnostic category, are outlined in table 1. Concentrated fluorescence smear microscopy was performed on NALC/NaOH processed sputum/non-sputum samples, and cultures were performed using the MGIT 960 liquid culture system (BD Diagnostics, USA).

**Table 1:** Demographic, clinical, sampling and microbiological characteristics of study patients stratified by TB diagnostic group.

	All	Definite TB	Probable TB	Non TB	Unclassified TB	P-value
	N = 281	N = 116	N = 71	N = 27	N = 67	
**Demographics**						
Median age (yrs, IQR)	35 (29–39)	35 (28–39)	33 (30–39)	34 (32–40)	36 (30–41)	n/s
Female	174 (62)	69 (59)	45 (63)	19 (70)	41 (61)	n/s
Median CD4 count (cells/ml, IQR)	89 (46–198)	86 (42–192)^*^	120 (51–215)^*^	128 (74–235)^*^	77 (42–120)^*^	^*^0.001
Previous TB	97 (35)	33 (28)^*^	22 (31)	11 (41)	31 (46)^*^	^*^0.01
Current Smoker	52 (19)	25 (22)	9 (13)	3 (11)	15 (22)	n/s
**Clinical features**						
Cough >2wks	231 (82)	99 (85)	58 (82)	22 (82)	52 (78)	n/s
Night sweats	185 (66)	82 (71)	49 (69)	14 (52)	40 (60)	n/s
Weight loss	247 (88)	105 (91)	61 (86)	22 (82)	59 (88)	n/s
Fever >38°C	49 (18)	29 (26)^* *1^	9 (13)^*^	3 (12)	8 (12)^*1^	^*^0.04^*1^0.03
CXR compatible with TB	215 (77)	99 (85)^* *1^	50 (70)^*^	16 (60)^*1^	50 (75)	^*^0.01^*1^0.002
Early Empiric Rx given	120 (43)	59 (51)	34 (48)	n/a	27 (40)	n/s
LAM strip test positive (grade 2)	98 (35)	58 (50)^*^	27 (38)^*1^	1 (4)	12 (18)^* *1^	^*^<0.001^*1^0.009
**Clinical samples collected for TB culture**						
1 sputum sample	207 (74)	95 (82)^*^	43 (61)^*^	21 (78)	48 (72)	^*^0.001
≥2 sputum samples	92 (33)	42 (26)	16 (23)	9 (33)	25 (37)	n/s
1 non-sputum sample	160 (57)	78 (67)^* *1^	43 (61)	11 (41)^*^	28 (42)^*1^	^*^0.01^*1^<0.001
≥2 non-sputum sample	56 (20)	26 (22)	14 (20)	3 (11)	13 (19)	n/s
No samples	19 (7)	0 (0)	5 (7)	4 (15)	10 (15)	n/s

P-values indicate significant differences between patient groups (marked with * and number to indicate comparison group) for different patient characteristics.

n/a: not applicable; n/s: not significant (p>0.05).

### TB Reference Standard and Case Definitions

The reference standard for definite-TB was liquid culture positivity for *Mycobacterium tuberculosis* from at least a single sample. Given the significant potential for misclassification bias due to challenges of sampling extra-pulmonary compartments, the significant proportion of sputum scarce patients, and the limited performance of a single liquid TB culture in HIV-infected patients [20], patients were further categorised into the following diagnostic groups for analysis (Figure 1):

#### Definite-TB

At least 1 *M. tuberculosis* sample positive by liquid culture (either sputum or non-sputum e.g. pleural fluid, pericardial fluid etc.).

#### Probable-TB

Not meeting the criterion for definite-TB but a clinical-radiological picture highly suggestive of TB. All patients in this group received and showed a good response to anti-TB treatment at two-month follow-up. Smear-positive but culture-negative or contaminated patient samples were included in this group.

#### Non-TB

No culture-based evidence of *M. tuberculosis* and an alternative diagnosis available. No clinical deterioration on two-month follow-up and no TB treatment given. Patients culture positive for non-tuberculosis mycobacteria (NTM) and not receiving anti-TB treatment were assigned to this group.

#### Unclassifiable TB

Unable to assign to any of the above-mentioned diagnostic groups due to death of unknown cause (without autopsy), on-going but uncharacterised symptoms at follow-up, or loss-to-follow-up at 2 months.

### Early Empiric Treatment Definition

In order to compare the diagnostic performance of the urine LAM strip testing with routine clinical practice, **early empiric treatment** was defined as any patient commencing TB treatment within 24 hours of hospital admission based only on clinical and/or radiological findings, and prior to the availability of any smear or culture results. All early empiric treatment decisions, even if initial made by junior staff (medical officers and registrars), were approved by the attending consultant general physician.

### Modelling Clinical Predictors Using Multiple Imputation

A univariate analysis was used to determined basic clinical, laboratory and radiological predictors of definite-TB. A set of multivariate clinical predictors was generated using stepwise logistic regression modeling. Multiple imputation by chained Equations (Royston, P & White, I 2011) was used to impute missing data prior to model building. The variables included in the logistic regression modeling included the following (number of missing data points for each variable that were imputated is indicated in the brackets): sex (2), age (5), previous TB history (0), known TB contact (0), current smoker (0), cough ≥2 weeks (0), productive cough (0), haemoptysis (0), self-reported weight loss (0), appetite loss (0), recent fever (0), night sweats (0), fatigue (0), shortness of breath (0), chest pain (0), abdominal pain (0), nausea/vomiting (0), diarrhea (0), neurological symptoms (0), measured weight (39), temperature (7), respiratory rate (7), and Modified early warning score (MEWS) (144) at enrollment, CXR compatibility with TB (24), and urine dipstick abnormalities (0). Different data appeared to be missing for different patients in a random fashion. The continuous variables (weight and temperature) were dichotomised using receiver operating characteristic (ROC) analysis to identify cut-points that maximised discriminatory utility prior to inclusion in the model. Rounded ß-coefficients from the reduced model of significant variables were used to generate scores to quantitate relevant clinical predictors. ROC analysis was performed and three cut-points were selected for rule-in, Youden’s index (the optimal mathematical balance between sensitivity and specificity [21]) and rule-out value. Diagnostic accuracy, including 95% confidence intervals, for each cut-point was assessed using sensitivity, specificity, positive and negative predictive values (PPV, NPV) and positive likelihood ratio (LR+).

### Urine Sampling and LAM Methodology

All patients gave a spot urine sample (10–30 ml) collected in a sterile container as soon as possible after recruitment. Urine was frozen on the day of collection and stored at −20°C for later batched testing. Urine LAM strip testing (Determine® TB, Alere, USA) was performed on thawed urine according to the manufacturer’s instructions by readers blinded to all patient data and reference test results. Urine LAM strip test lot#101102, the same as used for test evaluation in an outpatient ARV-clinic setting [18], was used. Detailed methodology for reading the urine LAM strip tests has been previously described [13]. Analysis was performed using the grade 2 cut-point which has shown better inter-observer reliability and good rule-in value (LR+ >10) in hospitalised HIV-infected patients [13].

### Statistical Analysis

Analyses were restricted to HIV infected patients only and were performed using definite- and probable- (combined) versus non-TB patient groups for the primary determination of diagnostic accuracy (unclassifiable patients were excluded). In addition, given the inability to accurately evaluate the specificity of empiric treatment in the primary analysis as treatment response formed part of the diagnostic categorisation, alternative analyses were performed (see online supplementary material) using either *M. tuberculosis* complex culture-positive versus negative groups, or only definite- versus non-TB groups. Diagnostic accuracy, including 95% confidence intervals, for individual tests and early empiric treatment was assessed using sensitivity, specificity and LR+. Given the variations in test specificity and very high study prevalence of TB, ranges of positive and negative predicative values are presented for individual tests and early empiric treatment at differing rates of in-patient TB prevalence. STATA IC, version 11 (Stata Corp, Texas, USA) was used for all statistical analyses.

## Results

### Demographics, Basic Clinical and TB Diagnostic Sampling Characteristics

Figure 1 outlines the study population. 16% (54/335) of enrolled patients were HIV uninfected and hence excluded from further analysis. 41% (116/281) of patients had definite-TB, an additional 25% (71/281) of patients had probable-TB, and only 10% (27/281) had non-TB. 24% (67/281) of patients remained unclassifiable due to death or lost-to-follow-up and were excluded from the primary analysis. Table 1 outlines demographics, basic clinical characteristics of the patient cohort and the sputum/non-sputum diagnostic samples stratified by TB diagnostic category. These same patient characteristics stratified by smear, culture and CD4 count have been previously described [13], [22]. The median (IQR) CD4 cell count of unclassifiable patients was 77 (42–120) cells/ml, similar to definite-TB patients [86 (42–192) cells/ml], but lower than either probable- [120 (51–215) cells/ml] or non-TB patients [128 (74–235) cells/ml] (p = 0.001). Compared to definite-TB patients, unclassifiable patients provided fewer non-sputum samples for TB culture [67% (78/116) vs. 42% (28/67), p<0.001].

### Routine Early Empiric Treatment Compared to the Urine LAM Strip Test

Table 2 compares the sensitivity (95% CI), specificity and LR+ of routine early empiric treatment, urine LAM strip test and CXR for definite- and probable-TB compared to non-TB patients (definite-TB to non-TB patients only compared in table S2). Early empiric treatment identified and the urine LAM strip test diagnosed an approximately equal number TB cases with sensitivities of 50% (43–57, 93/187) and 46% (39–53, 85/187) respectively (p = 0.4). The combined sensitivity of urine LAM strip testing together with early empiric treatment was higher than either modality alone [LAM and early empiric treatment: 71% (95% CI: 64–77, 132/187) vs. LAM alone: 46% (95% CI: 39–53, 85/187) vs. early empiric treatment alone: 50% (95% CI: 43–57, 93/187), both p<0.001]. In the primary analysis, both early empiric treatment and the urine LAM strip test showed specificities >95%. However, when an alternative analysis restricted to only patients with a valid *M. tuberculosis* culture result and comparing culture-positive and -negative groups is performed (results provided in table S1), although test sensitivities are not significantly lower, the specificities of both early empiric treatment and urine LAM strip testing decrease, with urine LAM offering higher specificity than early empiric treatment [75% (67–82, 95/126) vs. 63% (54–71, 79/126), p = 0.03]. Given this variable test specificity and high overall study TB prevalence, Table 3 presents a range of PPV (95% CI) and NPV values using three specificities for each diagnostic method (lowest, highest and average) at in-patient TB prevalence rates of 35%, 45%, and 55%, which could be expected to occur in the majority of endemic country hospital settings. The lowest specificities used in Table 3 are taken from the specificities presented in table S1, the highest from Table 2 and the third is an average of the highest and lowest. With an in-patient TB prevalence of 45% (*M. tuberculosis* culture positive TB prevalence in study = 48%, 116/242), the PPV ranges for early empiric treatment, urine LAM strip test and a combination thereof was 53–100%, 62–90% and 71–94%, respectively.

**Table 2:** Sensitivity, specificity and positive likelihood ratio of early empiric treatment, the urine LAM strip test, and CXR for TB diagnosis in hospitalised HIV-infected patients using the definite and probable-TB groups for sensitivity, and the non-TB groups for specificity analyses.

Diagnostic method	Sensitivity (%) (95% CI)	Specificity (%) (95% CI)	LR+ (95% CI)
**Early empiric Rx** †	50^*1*2^(43–57)93/187	100(88–100)27/27	N/C
**Urine LAM (grade 2 cut-point)**	46^*3*4^(39–53)85/187	96*5(82–99)26/27	12.3 (1.7–89.6)
**CXR**	85^*1*3^(79–89)159/187	30*5(16–49)8/27	1.2 (1.1–1.3)
**Early empiric Rx plus urine LAM** **(grade 2 cut-point)**	71^*2*4^(64–77)132/187	96(82–99)26/27	19.1 (2.7–136.1)

† Any patient commenced on TB treatment within 24 hours of hospital admission based only on clinical and radiological findings, and prior to the availability of any smear or culture results, is included in this group.

P-values indicate significant differences between tests (marked with * and number to indicate comparison group) for different diagnostic accuracy measures.

*1 p<0.001;

*2 p<0.001;

*3 p<0.001;

*4 p<0.001;

*5 p<0.001;

*6 p = 0.006.

LAM: Lipoarabinomannan; CXR: Chest X-ray; LR+: positive likelihood ratio; 95% CI: 95% Confidence interval; Rx: treatment.

**Table 3:** Calculated positive and negative predictive values for early empiric treatment, the urine LAM strip test and a combination thereof, using three data-generated estimates for test specificity and in-patient TB prevalence.

Diagnostic	Test sensitivity (%)	Test specificity (%)	In-patient TB prevalence					
			35%		45%		55%	
			PPV (%, 95 CI)	NPV (%, 95 CI)	PPV (%, 95 CI)	NPV (%, 95 CI)	PPV (%, 95 CI)	NPV (%, 95 CI)
Early Empiric Rx	51	63	43 (38–47)	71 (67–74)	53 (48–58)	61 (57–65)	63 (58–67)	51 (47–55)
	51	82	60 (55–66)	76 (72–79)	70 (65–75)	67 (64–71)	78 (73–82)	58 (54–62)
	50	100	100 (98–100)	79 (76–81)	100 (98–100)	71 (68–74)	100 (99–100)	63 (59–66)
Urine LAM strip test	50	75	52 (47–57)	74 (70–77)	62 (57–70)	65 (61–68)	71 (66–75)	55 (51–59)
	48	86	65 (59–70)	75 (72–78)	74 (68–78)	67 (63–70)	81 (76–85)	58 (54–61)
	46	96	86 (80–90)	77 (74–80)	90 (86–94)	69 (65–72)	93 (90–96)	60 (56–63)
**Early empiric Rx plus urine LAM (grade 2 cut–point)**	74	75	61 (57–66)	84 (81–87)	71 (67–75)	78 (74–81)	78 (75–82)	70 (66–74)
	73	86	74 (69–78)	86 (83–88)	81 (77–85)	80 (76–83)	87 (83–89)	72 (68–76)
	71	96	91 (87–94)	86 (83–88)	94 (90–96)	80 (77–83)	96 (93–97)	73 (69–77)

LAM: Lipoarabinomannan; PPV: positive predictive value; NPV: negative predictive value; 95% CI: 95% Confidence interval; Rx: treatment.

### Clinical Predictors Compared to the Urine LAM Strip Test

The univariate and multivariate associates of definite-TB are shown in Table S3. Table S4 shows the sensitivity (95% confidence intervals), specificity, and LR+ for ROC-selected cut-points, selected for their rule-in, rule-out, or best compromise between sensitivity and specificity (assuming equal weighting) for the quantified set of clinical predictors, the urine LAM strip test and early empiric treatment. At equivalent specificity, clinical predictors ≥2.5 had a lower sensitivity than urine LAM strip testing [10% (95% CI: 7–15, 19/187) vs. 45% (95% CI: 38–53, 85/187), p<0.001].

### Clinical Predictors and Early Empiric Treatment

42% (10/24) of patients ‘ruled-in’ by clinical predictors ≥2.5 and 38% (23/61) patients ‘ruled-out’ by clinical predictors ≤0.5 were given early empiric treatment by attending hospital clinicians. Table S5 provides a further comparison of patient characteristics for patients commencing vs. not commencing early empiric treatment. No differences in basic demographic, symptomatology or diagnostic sampling was noted between groups except that a higher proportion of patients given early empiric treatment had a cough >2 weeks [90% (108/120) vs. 76% (123/161), p = 0.003]. Patients given vs. not given early empiric treatment had a lower median MEWS [3 (1–5) vs. 4 (3–5), p = 0.001] and creatinine level [68 (56–94) vs. 77 (59–107) µmol/l, p = 0.04].

### Urine LAM Strip Test Positive Patients Missed by Early Empiric Treatment

**Figure 2:**
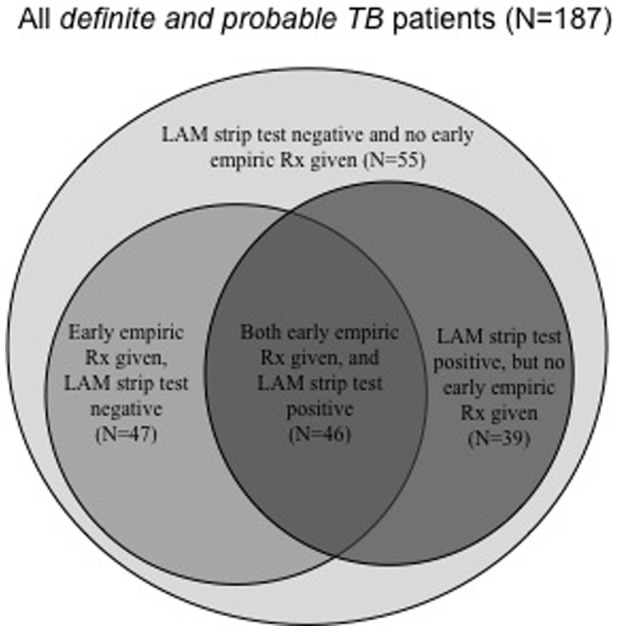
Venn diagram of all definite- and probable-TB patients indicating different but overlapping patient populations detected by the urine LAM strip test and early empiric TB treatment.

The Venn diagram in Figure 2 indicates the different but overlapping patient populations detected by urine LAM strip and early empiric treatment initiation. 21% (39/187) of definite- and probable-TB cases were urine LAM strip test positive, but missed by early empiric treatment. 64% (25/39) of these patients were either sputum smear-negative or unable to produce sputum. Table 4 compares the characteristics of definite- and probable-TB patients detected by either urine LAM alone, early empiric treatment or missed by both modalities. Patients detected by urine LAM alone, when compared to those detected by early empiric treatment alone, had a lower median (IQR) CD4 cell count [73 (31–134) vs. 138 (60–217), p = 0.008], a higher median (IQR) MEWS [5 (3–6) vs. 3 (1–4), p = 0.001] and an increased urea level [5.6 (4.0–11.6) vs. 3.7 (3.2–4.6), p = 0.002]. Patients detected by urine LAM alone, when compared to those missed by both urine LAM and early empiric treatment, had lower median CD4 cell count [73 (31–134) vs. 173 (58–269) units, p = 0.003)]. If the analysis was repeated using only definite-TB patients the findings were similar (Figure S1) and conclusions unchanged. 24% (28/116) of culture-positive patients were LAM positive but missed by early empiric treatment. When compared to the 23% (27/116) of patients that were LAM-negative but started on early empiric treatment, they had a lower median (IQR) CD4 cell count [62 (31–110) vs. 107 (54–171) cells/ml, p = 0.05], a higher median (IQR) MEWS [5 (3–6) vs. 3 (1–4), p = 0.01] and an increased urea level [5.5 (3.7–11.2) vs. 3.7 (3.4–4.6), p = 0.02]. Patients detected by urine LAM alone had lower median (IQR) CD4 cell count and a higher 8-week mortality than those patients missed by both urine LAM and early empiric treatment [CD4 count: 62 (31–110) vs. 180 (74–308), p = 0.002; 8-week mortality: 24% (6/25) vs. 4% (1/23), p = 0.05].

**Table 4:** Comparison of patient characteristics between groups detected by either the urine LAM strip or early empiric treatment alone, or missed both bedside diagnostic methods (only patients with definite- or probable-TB were included).

Patient characteristic(s)	LAM strip positive, but no early empiric Rx given	LAM strip negative, but early empiric Rx given	LAM strip negative and no early empiric treatment given	†P-value
	(N = 39)	(N = 47)	(N = 55)	
	Median (IQR) or n(%)	Median (IQR) or n(%)	Median (IQR) or n(%)	
Age, years	32 (26–38)	35 (28–38)	35 (30–40)	n/s
Previous TB	12 (31)	16 (34)	18 (33)	n/s
Weight, kg	52 (47–60)	56 (44–63)	52 (47–61)	n/s
Temperature, °C	36.9 (36.0–37.1)	36.9 (36.5–38.0)	36.9 (36.3–37.7)	n/s
Respiratory rate, breaths/min	24 (20–28)	22 (19–29)	24 (18–28)	n/s
CD4 cell count, cells/ml	73 (31–134)^* #^	138 (60–217)^*^	173 (58–269)^#^	^*^0.008 ^#^0.003
MEWS§	5 (3–6)^*^	3 (1–4)^*^	4 (3–6)	^*^0.001
Urea, mmol/l	5.6 (4.0–11.6)^*^	3.7 (3.2–4.6)^*^	4.2 (3.2–7.8)	^*^0.002
Creatinine, µmol/l	95 (59–121)^*^	67 (55–79)^*^	74 (57–102)	^*^0.01
8-week mortality	6/36 (17)	3/40 (8)	3/39 (8)	n/s

§ MEWS: Modified early warning score is an admission triage score based on illness severity and higher scores correlated with poor outcomes and increased mortality [19].

† P-values indicate significant differences between patient groups (marked with * and ^#^ to indicate comparison group) for different patient characteristics.

LAM: Lipoarabinomannan; Rx: treatment; IQR: interquartile range.

## Discussion

The point-of-care urine LAM test has potential as a useful adjunct for rapid TB diagnosis in HIV-infected hospitalised patients [11], [13]. Its added clinical value, however, remains uncertain given its modest performance characteristics. The key finding of this study is that LAM detected patients that would have otherwise been missed by empiric treatment and this subgroup of patients had more advanced immunosuppression and greater illness severity. The latter represents a group most likely to benefit from the initiation of early treatment as they are at high risk.

Traditional and newer TB diagnostics show reduced diagnostic accuracy in hospitalised co-infected patients, particularly with advanced immunosuppression, as patients are often unable to produce sputum for diagnostic testing and/or have disseminated disease [3]. In addition, these patients present to hospitals with late stage disease and severe illness [3]. These factors mean that treatment decisions are commonly made based on clinical and radiological findings alone, the need for urgent treatment initiation, and the high background disease prevalence (pre-test probability). Yet, in this same patient group, clinical and radiological findings are frequently atypical and/or non-specific, and this accounts for the poor rule-in value of the set of clinical predictors that we derived. Indeed, as a ‘rule-in’ test (cut-point selecting high specificity and PPV) clinical predictors could only correctly classify ∼20% of all patients. This assumes coherent mathematical analysis of available diagnostic variables. However, routine clinical decision-making is rather a dynamic Bayesian process of assimilating an accumulating series of pre-test probabilities [23], and weighting of the overall post-test probability of disease against a threshold probability for initiating treatment. Thus, in reality physician practice varies widely and in an attempt to reduce mortality hospital treatment thresholds are lower than expected. Indeed, in this study, ∼50% of definite- and probable-TB patients initiated early empiric TB treatment.

Early empiric TB treatment decisions should logically be targeted to the sickest patients, especially amongst hospitalised HIV-infected patients with advanced immunosuppression where given higher mortality rates, treatment-initiation thresholds should be lowered. However, this was not the case in those empirically treated. In fact overall, patients not commenced on early empiric treatment appeared, using the MEWS, to have a higher illness severity. No clear demographic, clinical or radiological factor predicted early empiric treatment. By contrast, the urine LAM strip test could pinpoint the most severely ill patients. Thus, our data suggest that early empiric treatment will miss a particularly vulnerable patient group with advanced immunosuppression that would have been detected by LAM strip testing. The rapid identification of these patients could target prompt therapy to those most likely to benefit. Recent studies with both the TB LAM ELISA and the LAM strip test support our findings, demonstrating associations between urine LAM positivity, higher mycobacterial disease burden, more advanced immunosuppression, and increased illness severity and mortality [15]–[17]. These findings support the need to undertake prospective impact studies to assess whether initiation of TB treatment based on urine LAM testing is able to save lives and/or decrease TB-related morbidity.

Do our findings have relevance to in-patient settings with a lower TB prevalence? Amongst in-patient settings with a lower TB prevalence, urine LAM is likely to offer superior ‘rule-in’ utility compared to empiric treatment. This is evidenced by: i) the poor comparative diagnostic utility and inferiority of a set of clinical predictors, which estimates pre-test probability or what could be expected with a frequentist interpretation of simple clinical and radiological predictors, and ii) lack of clear demographic, clinical or radiological parameters associated with early empiric treatment practice and limited agreement with the derived set of clinical predictors indicating the lack of predictability and hence, standardisation of empiric treatment decision-making. Given the modest performance characteristics of the urine LAM strip testing it is however clear that both urine LAM alone, or combined with existing empiric treatment practise, is likely to only offer clinically useful ‘rule-in’ utility (PPV>90%) in hospital settings with high TB prevalence (>35%).

This is the first study to compare the value of urine LAM strip testing against clinical-radiological screening and day-to-day clinical practice (early empiric treatment rates) in hospitalised patients. However, our study has important limitations. Given the well-established misclassification bias that occurs due to the drawbacks of the TB culture technique and the lack of interventional lung sampling (sputum induction and bronchoscopy), a diagnostic categorisation was used to group patients for analysis. This may have underestimated sensitivity and over-estimated specificity. The TB prevalence in our study was higher than in many other settings and this limits the generalisability of our findings. However, we have presented predictive values using estimated low, medium and high TB prevalence rates to improve generalisability. The study had a high proportion of unclassifiable-TB patients due to death and loss-to-follow-up, and these patients had a higher proportion of LAM strip test negative results than definite- and probable-TB groups. This may have introduced the possibility of selection bias, however, in our secondary analyses presented in the online supplementary materials we compare *M. tuberculosis* culture positive vs. negative groups and include unclassifiable-TB patients with a valid culture result. Key study findings are unaffected. Our study did not evaluate LAM against newer diagnostic standards such as the Xpert MTB/RIF assay. However, this was not accessible to us at the time of the study and this test offers reduced utility in extra-pulmonary and sputum scare TB. Urine LAM test results were not performed at the bedside or used to guide treatment initiation, thus a survival benefit through initiating early treatment in these severely ill patients is unclear but possible [8].

An ideal point-of-care test for rapid, laboratory-free detection of TB remains elusive [24]. However sampling hurdles and poor performance when using extra-pulmonary samples mean that the need to make empiric treatment decisions is likely to continue. Thus, despite only modest diagnostic accuracy, the low cost urine LAM strip test offers important added clinical value in hospitalised HIV-infected patients with suspected TB. Not only could the test detect patients missed by clinical and radiological predictors but also could potentially enable the rapid treatment of patients with the most advanced immunosuppression and severe illness. Further studies are now required to confirm our study findings and evaluate the impact of urine LAM strip testing to guide early treatment initiation in hospitalised HIV-infected patients.

## Supporting Information

Figure S1 Venn diagram of all definite-TB patients indicating different but overlapping patient populations detected by the urine LAM strip test and early empiric TB treatment.
(TIFF)

Table S1 Diagnostic accuracy measures of early empiric treatment, the urine LAM strip test and CXR for TB diagnosis in hospitalised HIV-infected patients using *M. tuberculosis* culture positive-TB patients for sensitivity, and culture negative patients for specificity analyses. ^§^All patients with 1 or more valid *M. tuberculosis* culture (either sputum or non-sputum) are included in this analysis irrespective of final TB diagnostic categorization (39/281 patients excluded with either no/contaminated culture result) ^†^Any patient commenced on TB treatment within 24 hours of hospital admission based only on clinical and radiological findings, and prior to the availability of any smear or culture results, is included in this group. P-values indicate significant differences between tests (marked with * and number to indicate comparison group) for different diagnostic accuracy measures ^*1^p<0.001; ^*2^p<0.001; ^*3^p<0.001; ^*4^p<0.001; ^*5^p = 0.03; ^*6^p = 0.03; ^*7^p<0.001;^ *8^p = 0.005.
(DOCX)

Table S2 Diagnostic accuracy measures of early empiric treatment, the urine LAM strip test and CXR for TB diagnosis in hospitalized HIV-infected patients using definite-TB (*M. tuberculosis* culture positive) for sensitivity, and non-TB patient groups for specificity analyses. ^†^Any patient commenced on TB treatment within 24 hours of hospital admission based only on clinical and radiological findings, and prior to the availability of any smear or culture results, is included in this group. P-values indicate significant differences between tests (marked with * and number to indicate comparison group) for different diagnostic accuracy measures ^*1^p<0.001; ^*2^p<0.001; ^*3^p<0.001; ^*4^p<0.001; ^*5^p<0.001.
(DOCX)

Table S3 Univariate and multivariate analyses for associates of definite-TB in HIV-infected hospitalised patients. ^†^Receiver operating characteristic (ROC) curve-selected cut-point maximizing discriminatory utility used to dichotomise the continuous variables weight and temperature OR: odds ratio; TB: Tuberculosis; CXR: Chest x-ray; LAM: Lipoarabinomannan.
(DOCX)

Table S4 Diagnostic accuracy measures for set of clinical predictors (using three ROC-selected cut-points), the urine LAM strip test and routine early empiric treatment in hospitalised HIV-infected patients using the definite and probable-TB groups for sensitivity and the non-TB groups for specificity analyses. P-values indicate significant differences between tests and/or cut-points (marked with * and number to indicate comparison group) for different diagnostic accuracy measures; ^*1^p<0.001; ^*2^p = 0.03**^†^** Youden’s index is defined as the point on the ROC curve that provides the optimal mathematical balance between sensitivity and specificity.
(DOCX)

Table S5 Demographic, clinical, sampling and microbiological characteristics of study patients stratified by TB diagnostic group ^†^ Any patient commenced on TB treatment within 24 hours of hospital admission based only on clinical and radiological findings, and prior to the availability of any smear or culture results, is included in this group. Analysis is performed for all patients in this graph and hence includes 27 unclassified patients whom were commenced on early empiric treatment but do not form part of the primary analysis presented in the main manuscript. P-values indicate significant differences between patient groups (marked with * and number to indicate comparison group) for different patient characteristics ^§^MEWS: Modified early warning score is an admission triage score based on illness severity and higher scores correlated with poor outcomes and increased mortality [19].
(DOCX)
