# Supplementary material for: Can Point-of-Care Urine LAM Strip Testing for Tuberculosis Add Value to Clinical Decision Making in Hospitalised HIV-Infected Persons?
Source: PLoS One. 2013 Feb 4;8(2):e54875. doi: 10.1371/journal.pone.0054875 (PMC3563660; doi:10.1371/journal.pone.0054875)
Supplement: Table S1 — Diagnostic accuracy measures of early empiric treatment, the urine LAM strip test and CXR for TB diagnosis in hospitalised HIV-infected patients using M. tuberculosis culture positive-TB patients for sensitivity, and culture negative patients for specificity analyses. §All patients with 1 or more valid M. tuberculosis culture (either sputum or non-sputum) are included in this analysis irrespective of final TB diagnostic categorization (39/281 patients excluded with either no/contaminated culture result) †Any patient commenced on TB treatment within 24 hours of hospital admission based only on clinical and radiological findings, and prior to the availability of any smear or culture results, is included in this group. P-values indicate significant differences between tests (marked with * and number to indicate comparison group) for different diagnostic accuracy measures *1p<0.001; *2p<0.001; *3p<0.001; *4p<0.001; *5p = 0.03; *6p = 0.03; *7p<0.001; *8p = 0.005. (DOCX) [file pone.0054875.s002.docx]

**Table S1**. Diagnostic accuracy measures of early empiric treatment, the urine LAM strip test and CXR for TB diagnosis in hospitalised HIV-infected patients using *M. tuberculosis* culture positive-TB patients for sensitivity, and culture negative patients for specificity analyses.

| **Diagnostic method** | **Sensitivity (%) (95% CI)** | **Specificity (%) (95% CI)** | **PPV (%) (95% CI)** | **NPV (%) (95% CI)** | **LR+ (95% CI)** |
| --- | --- | --- | --- | --- | --- |
| **Early empiric Rx^†^** | 51^*1*2^ (42-60) 59/116 | 63^*5*6*7^ (54-71) 79/126 | 56^*8^ (46-65) 59/106 | 58 (50-66) 79/136 | 1.36 (1.27-1.47) |
| **Urine LAM (grade 2 cut-point)** | 50^*3*4^ (41-59) 58/116 | 75^*5^ (67-82) 95/126 | 65 (55-74) 58/89 | 63 (57-69) 95/153 | 2.03 (1.84-2.24) |
| **CXR** | 92^*1*3^ (86-96) 107/116 | 21^*7^ (15-29) 27/126 | 52 (45-59) 107/206 | 75 (58-86) 27/36 | 1.17 (1.15-1.20) |
| **Early empiric Rx plus urine LAM (grade 2 cut-point)** | 74^*2*4^ (66-81) 86/116 | 75^*6^ (67-82) 95/126 | 74^*8^ (65-81) 86/117 | 76 (68-83) 95/125 | 3.01 (2.81-3.24) |

^§^All patients with 1 or more valid *M. tuberculosis* culture (either sputum or non-sputum) are included in this analysis irrespective of final TB diagnostic categorization (39/281 patients excluded with either no/contaminated culture result)

^†^Any patient commenced on TB treatment within 24 hours of hospital admission based only on clinical and radiological findings, and prior to the availability of any smear or culture results, is included in this group.

P-values indicate significant differences between tests (marked with * and number to indicate comparison group) for different diagnostic accuracy measures

^*1^p<0.001; ^*2^p<0.001; ^*3^p<0.001; ^*4^p<0.001; ^*5^p=0.03; ^*6^p=0.03; ^*7^p<0.001; ^*8^p=0.005
