# Supplementary material for: Can Point-of-Care Urine LAM Strip Testing for Tuberculosis Add Value to Clinical Decision Making in Hospitalised HIV-Infected Persons?
Source: PLoS One. 2013 Feb 4;8(2):e54875. doi: 10.1371/journal.pone.0054875 (PMC3563660; doi:10.1371/journal.pone.0054875)
Supplement: Table S2 — Diagnostic accuracy measures of early empiric treatment, the urine LAM strip test and CXR for TB diagnosis in hospitalized HIV-infected patients using definite-TB (M. tuberculosis culture positive) for sensitivity, and non-TB patient groups for specificity analyses. †Any patient commenced on TB treatment within 24 hours of hospital admission based only on clinical and radiological findings, and prior to the availability of any smear or culture results, is included in this group. P-values indicate significant differences between tests (marked with * and number to indicate comparison group) for different diagnostic accuracy measures *1p<0.001; *2p<0.001; *3p<0.001; *4p<0.001; *5p<0.001. (DOCX) [file pone.0054875.s003.docx]

**Table S2**. Diagnostic accuracy measures of early empiric treatment, the urine LAM strip test and CXR for TB diagnosis in hospitalized HIV-infected patients using definite-TB (*M. tuberculosis* culture positive) for sensitivity, and non-TB patient groups for specificity analyses.

| **Diagnostic method** | **Sensitivity (%) (95% CI)** | **Specificity (%) (95% CI)** | **PPV (%) (95% CI)** | **NPV (%) (95% CI)** | **LR+ (95% CI)** |
| --- | --- | --- | --- | --- | --- |
| **Early empiric Rx^†^** | 51^*1*2^ (42-60) 59/116 | 100 (88-100) 27/27 | 100 (94-100) 59/59 | 32 (23-43) 27/84 | N/C |
| **Urine LAM (grade 2 cut-point)** | 50^*3*4^ (41-59) 58/116 | 96^*5^ (82-99) 26/27 | 98 (91-100) 58/59 | 31 (22-42) 26/84 | 13.5 (1.8-99.1) |
| **CXR** | 92^*1*3^ (86-96) 107/116 | 30^*5^ (16-49) 8/27 | 85 (78-90) 107/126 | 47 (26-69) 8/17 | 1.3 (1.2-1.5) |
| **Early empiric Rx plus urine LAM (grade 2 cut-point)** | 74^*2*4^ (66-81) 86/116 | 96 (82-99) 26/27 | 99 (94-100) 86/87 | 46 (34-59) 26/56 | 20.0 (2.8-143.2) |

^†^Any patient commenced on TB treatment within 24 hours of hospital admission based only on clinical and radiological findings, and prior to the availability of any smear or culture results, is included in this group.

P-values indicate significant differences between tests (marked with * and number to indicate comparison group) for different diagnostic accuracy measures

^*1^p<0.001; ^*2^p<0.001; ^*3^p<0.001; ^*4^p<0.001; ^*5^p<0.001
