# Supplementary material for: Can Point-of-Care Urine LAM Strip Testing for Tuberculosis Add Value to Clinical Decision Making in Hospitalised HIV-Infected Persons?
Source: PLoS One. 2013 Feb 4;8(2):e54875. doi: 10.1371/journal.pone.0054875 (PMC3563660; doi:10.1371/journal.pone.0054875)
Supplement: Table S3 — Univariate and multivariate analyses for associates of definite-TB in HIV-infected hospitalised patients. †Receiver operating characteristic (ROC) curve-selected cut-point maximizing discriminatory utility used to dichotomise the continuous variables weight and temperature OR: odds ratio; TB: Tuberculosis; CXR: Chest x-ray; LAM: Lipoarabinomannan. (DOCX) [file pone.0054875.s004.docx]

**Table S3**. Univariate and multivariate analyses for associates of definite-TB in HIV-infected hospitalised patients.

| Patient characteristic | Univariate analysis | | | |
| --- | --- | --- | --- | --- |
|  | OR (95% CI) | P-value | β-coefficient | Score |
| Previous TB | 0.57 (0.33-0.97) | 0.04 | n/a | n/a |
| Weight | 0.97 (0.95-0.99) | 0.01 | n/a | n/a |
| Weight ≤50 kg^†^ | 2.1 (1.2-3.7) | 0.008 | n/a | n/a |
| Temperature | 1.5 (1.1-2.0) | 0.005 | n/a | n/a |
| Temperature ≥37.5 ºC^†^ | 2.6 (1.4-4.7) | 0.002 | n/a | n/a |
| CXR potentially TB | 3.0 (1.4-6.9) | 0.007 | n/a | n/a |
| LAM Ag rapid test (cutpoint1) | 3.8 (2.2-6.4) | <0.001 | n/a | n/a |
| Multivariate analysis (clinical and radiology predictors only) | | | | |
| History of previous TB | 0.52 (0.29-0.93) | 0.03 | -0.65 | -0.5 |
| Weight ≤ 50 kg | 2.5 (1.4-4.6) | 0.002 | 0.92 | 1 |
| Temperature ≥ 37.5 ºC | 2.8 (1.5-5.2) | 0.001 | 1.02 | 1 |
| CXR compatible with TB | 3.0 (1.3-7.0) | 0.01 | 1.09 | 1 |

^†^Receiver operating characteristic (ROC) curve-selected cut-point maximizing discriminatory utility used to dichotomise the continuous variables weight and temperature

OR: odds ratio; TB: Tuberculosis; CXR: Chest x-ray; LAM: Lipoarabinomannan
