# Supplementary material for: Can Point-of-Care Urine LAM Strip Testing for Tuberculosis Add Value to Clinical Decision Making in Hospitalised HIV-Infected Persons?
Source: PLoS One. 2013 Feb 4;8(2):e54875. doi: 10.1371/journal.pone.0054875 (PMC3563660; doi:10.1371/journal.pone.0054875)
Supplement: Table S4 — Diagnostic accuracy measures for set of clinical predictors (using three ROC-selected cut-points), the urine LAM strip test and routine early empiric treatment in hospitalised HIV-infected patients using the definite and probable-TB groups for sensitivity and the non-TB groups for specificity analyses. P-values indicate significant differences between tests and/or cut-points (marked with * and number to indicate comparison group) for different diagnostic accuracy measures; *1p<0.001; *2p = 0.03† Youden’s index is defined as the point on the ROC curve that provides the optimal mathematical balance between sensitivity and specificity. (DOCX) [file pone.0054875.s005.docx]

**Table S4.** Diagnostic accuracy measures for set of clinical predictors (using three ROC-selected cut-points), the urine LAM strip test and routine early empiric treatment in hospitalised HIV-infected patients using the definite and probable-TB groups for sensitivity and the non-TB groups for specificity analyses.

| **Type of TB detection test** | **Cut-point value** | **Sensitivity** | **Specificity** | **LR+** |
| --- | --- | --- | --- | --- |
|  |  | **(%)** | **(%)** |  |
|  |  | **(95% CI)** | **(95% CI)** | **(95% CI)** |
| **Urine LAM** |  | 46^*1^ | 96 |  |
|  | **Grade 2** | (39-53) | (92-99) | 12.3 |
|  |  | 85/187 | 26/27 | (1.7-89.6) |
|  |  | 50^*1*2^ | 100 |  |
| **Early empiric Rx^†^** | **n/a** | (43-57) | (88-100) | N/C |
|  |  | 93/187 | 27/27 |  |
| **Quantified set of clinical predictors** | | | | |
| **‘rule-out’** |  | 91 | 15 |  |
|  | **≥ 0.5** | (87-95) | (6-33) | 1.07 |
|  |  | 171/187 | 4/27 | (0.99-1.17) |
| **Youden’s index^†^** |  | 57 | 67 |  |
|  | **≥ 1.5** | (50-64) | (48-81) | 1.7 |
|  |  | 106/187 | 18/27 | (1.35-2.14) |
| **‘rule-in’** |  | 10^*1^ | 100 |  |
|  | **≥ 2.5** | (7-15) | (88-100) | N/C |
|  |  | 19/187 | 27/27 |  |

P-values indicate significant differences between tests and/or cut-points (marked with * and number to indicate comparison group) for different diagnostic accuracy measures; ^*1^p<0.001; ^*2^p=0.03**^†^** Youden’s index is defined as the point on the ROC curve that provides the optimal mathematical balance between sensitivity and specificity
