# Supplementary material for: Can Point-of-Care Urine LAM Strip Testing for Tuberculosis Add Value to Clinical Decision Making in Hospitalised HIV-Infected Persons?
Source: PLoS One. 2013 Feb 4;8(2):e54875. doi: 10.1371/journal.pone.0054875 (PMC3563660; doi:10.1371/journal.pone.0054875)
Supplement: Table S5 — Demographic, clinical, sampling and microbiological characteristics of study patients stratified by TB diagnostic group † Any patient commenced on TB treatment within 24 hours of hospital admission based only on clinical and radiological findings, and prior to the availability of any smear or culture results, is included in this group. Analysis is performed for all patients in this graph and hence includes 27 unclassified patients whom were commenced on early empiric treatment but do not form part of the primary analysis presented in the main manuscript. P-values indicate significant differences between patient groups (marked with * and number to indicate comparison group) for different patient characteristics §MEWS: Modified early warning score is an admission triage score based on illness severity and higher scores correlated with poor outcomes and increased mortality [19]. (DOCX) [file pone.0054875.s006.docx]

**Table S5.** Demographic, clinical, sampling and microbiological characteristics of study patients stratified by TB diagnostic group

|  | **All** | **Early empiric Rx given^†^** | **No early empiric Rx** | **P-value** |
| --- | --- | --- | --- | --- |
|  | **N=281** | **N=120** | **N=161** |  |
| **Demographics** |  | | | |
| Median age (yrs, IQR) | 35 (29-39) | 35 (30-40) | 34 (29-39) | n/s |
| Female (n, %) | 174 (62) | 69 (58) | 105 (65) | n/s |
| Median CD4 count (cells/ml) | 86 (46-198) | 84 (47-171) | 93 (45-212) | n/s |
| Previous TB (n, %) | 97 (35) | 42 (35) | 55 (34) | n/s |
| Current Smoker (n, %) | 52 (19) | 26 (22) | 26 (16) | n/s |
| **Clinical features** |  | | | |
| Cough >2wks (n, %) | 231 (82) | 108 (90)^*^ | 123 (76)^*^ | ^*^0.003 |
| Night sweats (n, %) | 185 (66) | 77 (64) | 108 (67) | n/s |
| Self-reported Weight loss (n, %) | 247 (88) | 104 (87) | 143 (89) | n/s |
| Fever >38°C (n, %) | 49 (18) | 22 (19) | 27 (17) | n/s |
| Median weight (kgs, IQR) | 53 (47-63) | 52 (45-62) | 53 (48-63) | n/s |
| Median temperature (°C, IQR) | 36.8 (36.1-37.5) | 36.9 (36.2-37.5) | 36.8 (36.1-37.5) | n/s |
| Respiratory rate (breaths/min, IQR) | 22 (19-28) | 23 (20-28) | 22 (19-28) | n/s |
| Median MEWS^§^ (IQR) | 4 (2-5) | 3 (1-5)^*^ | 4 (3-5)^*^ | 0.001 |
| Median urea (mmol/l, IQR) | 4.8 (3.5-8) | 4.5 (3.6-7.5) | 5.3 (3.5-8.9) | n/s |
| Median creatinine (µmol/l, IQR) | 72.5 (57-100) | 68 (56-94)^*^ | 77 (59-107)^*^ | 0.04 |
| CXR compatible with TB (n, %) | 215 (77) | 98 (82) | 117 (73) | n/s |
| LAM strip test positive (grade 2) (n, %) | 98 (35) | 50 (42)^*^ | 48 (30)^*^ | ^*^0.04 |
| **Clinical samples collected for TB culture** | | | | |
| 1 sputum sample (n, %) | 207 (74) | 91 (76) | 116 (72) | n/s |
| ≥2 sputum samples (n, %) | 92 (33) | 35 (29) | 57 (35) | n/s |
| 1 non-sputum sample (n, %) | 160 (57) | 67 (56) | 93 (58) | n/s |
| ≥2 non-sputum sample (n, %) | 56 (20) | 24 (20) | 32 (20) | n/s |
| No samples (n, %) | 19 (7) | 5 (4)^*^ | 14 (8) | n/s |

^†^ Any patient commenced on TB treatment within 24 hours of hospital admission based only on clinical and radiological findings, and prior to the availability of any smear or culture results, is included in this group. Analysis is performed for all patients in this graph and hence includes 27 unclassified patients whom were commenced on early empiric treatment but do not form part of the primary analysis presented in the main manuscript.

P-values indicate significant differences between patient groups (marked with * and number to indicate comparison group) for different patient characteristics

^§^MEWS: Modified early warning score is an admission triage score based on illness severity and higher scores correlated with poor outcomes and increased mortality [[19](#_ENREF_19)]
